# Anterior Lung Evisceration Following an Assault with Knife: A Case Report

**DOI:** 10.5811/cpcem.2021.4.51603

**Published:** 2021-07-27

**Authors:** Martín Ferreira-Pozzi, Pablo Joaquin Erramouspe, Juan Carlos Folonier, Mauro Perdomo Perez, Daniel González González, Erik G. Laurin

**Affiliations:** * University of the Republic, Maciel hospital, Department of Surgery, Clínica quirúrgica 3, Montevideo, Uruguay; † Queensland University of Technology, Translational Research Institute, Brisbane, Queensland, Australia; ‡ University of California Davis Health, Department of Emergency Medicine, Sacramento, California

**Keywords:** Penetrating injury, open trauma, chest trauma, lung hernias, lung evisceration

## Abstract

**Introduction:**

Evisceration of the lung is a rare consequence of open chest trauma that can be fatal. Evisceration of the lung refers to the protrusion of lung parenchyma through a defect of the thoracic wall, without parietal pleural or skin coverage.

**Case report:**

A 20-year-old man was brought to the emergency department (ED) with left lung evisceration from stab wounds. The eviscerated lung was left in place, and the patient was not intubated in the ED. He was immediately taken to the operating room (OR) for intubation and surgical repair. Other significant injuries were ruled out, the eviscerated lung was retrieved, the chest wall defect was closed, and the patient recovered well. He was discharged after seven days in good condition.

**Conclusion:**

The initial management of patients with lung evisceration is critical to prevent rapid decompensation and death. Appropriate ED airway management, lung retrieval in the OR, and thoracic wall repair is recommended for patients with lung evisceration.

## INTRODUCTION

Evisceration of the lung is an uncommon consequence of chest trauma that can lead to respiratory distress and death. This condition requires an accurate assessment of the patient’s respiratory condition and early definitive surgical repair. Without appropriate medical treatment patients with this medical and surgical pathology would not survive to the operating room for surgical treatment. Unfortunately, there are few articles with recommendations on the medical management of these patients.^1^–^6^ Moreover, there is often confusion regarding the terminology used to define evisceration. Among clinicians, evisceration and herniation of the lung are terms sometimes used interchangeably. However, it is important to highlight that these two entities are quite different pathologies. Evisceration of the lung refers to the protrusion of parenchyma through a defect of the thoracic wall without parietal pleura or skin coverage.^1^ On the other hand, lung herniation is the protrusion of the lung parenchyma through the chest wall, which is covered by skin or pleura.

Lung herniation has a more common and less dramatic presentation than evisceration.^2^,^7^ Lung hernias are classified based on the etiology and anatomy, and two etiologic groups are recognized: congenital and acquired, the latter further divided into traumatic or spontaneous. Congenital hernias are caused by attenuation of the endothoracic fascia, occurring either at the thoracic inlet or an intercostal space where weakness of the fascia is usually combined with congenital absence of intercostal muscles.^8^ On the other hand, acquired hernias are caused by weakness of the intercostal muscles in combination with a sudden increase of intrathoracic pressure, usually seen as complications after thoracotomies. This could be one of the reasons why the two entities are sometimes confused, as thoracotomies are used during the repair of lung eviscerations yet may lead to complications such as lung herniation.^9^ The most common cause of acquired lung hernia is high-energy trauma to the chest, either penetrating or blunt, particularly following a motor vehicle accident.^10^ Additionally, acquired hernias have been reported after traumatic cardiopulmonary resuscitation with multiple rib fractures.^11^

The mechanism responsible for evisceration of the lung also involves high-energy trauma but is typically associated with penetrating thoracic injuries.^2^ Stab wounds are a common mechanism for this injury since the defect in the chest wall can be large enough for lung to eviscerate.^1^,^4^,^6^,^7^ Blunt trauma may also be a mechanism, with displaced rib fractures of the chest wall and elevated intrathoracic pressure causing fractured ribs to pierce the parietal pleura and skin. Pneumothorax may or may not be present, and hemorrhage from the internal thoracic artery has been described.^1^,^3^

In contrast to lung eviscerations, the clinical diagnosis of lung hernias can be challenging because symptoms, in addition to pain, can be subtle such as a subcutaneous mass and breath-dependent expansion of the mass. In these scenarios, a radiograph can be suggestive, but an early computed tomography (CT) is recommended for correct diagnosis.^12^ The diagnosis of lung evisceration, however, is usually obvious but the presentation may vary. While some patients may present in stable condition, others may have respiratory distress or arterial hemorrhage. Hernias and evisceration also differ in management. While small hernias do not typically require advanced airway management, lung evisceration often requires endotracheal intubation and operative care. Finally, some hernias can be managed with a nonsurgical approach, but surgical repair is recommended for large hernias and eviscerations.^13^,^14^

## CASE REPORT

**Image 1:**
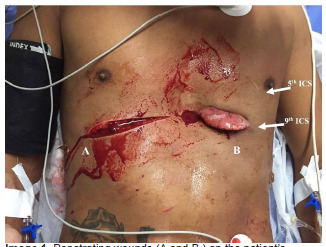
Penetrating wounds (A and B) on the patient’s right and left anterior chest during primary assessment in the emergency department. *ICS*, intercostal space.

A 20-year-old man with no remarkable past medical history was brought to the emergency department (ED) of a resource-limited hospital by ambulance with bilateral, anterior thoracic stab-wound lacerations (Image 1).

**Image 2:**
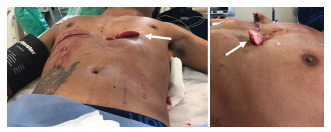
The lingula of the lung can be observed protruding beyond the penetrating injury of the thorax, expanding with each inspiration (arrows).

The patient was awake and responsive with a Glasgow Coma Scale of 15; he had a heart rate of 84 beats per minute, blood pressure of 90/60 millimeters of mercury, 22 breaths per minute, and oxygen saturation of 97% on room air. He complained of bilateral chest pain and shortness of breath. Physical exam primary survey revealed decreased breath sounds on the left, with bilateral large anterior chest wall lacerations. The left chest laceration had lung parenchyma protruding, and it expanded with each inspiration (Image 2). There were no other traumatic injuries or active hemorrhage on further primary survey, and secondary survey was unremarkable.

Closer examination of the wounds showed the right chest wound at the ninth intercostal space (ICS) with no evidence of exposed lung, and the left chest wound also at the ninth ICS but clearly exposing lung parenchyma. Bedside focused assessment with sonography for trauma (FAST) did not demonstrate a pericardial effusion or intra-abdominal free fluid. The standard blood tests for trauma patients were collected in this patient and were unremarkable, including initial hemoglobin of 13.6 grams (g) per deciliter (dL) (reference range: 12.0 – 16.0 g/dL).

**CPC-EM Capsule**

What do we already know about this clinical entity?

*Evisceration of the lung is a rare consequence of open chest trauma that can be fatal*.

What makes this presentation of disease reportable?

*This case demonstrates that basic airway management was enough to maintain proper levels of oxygenation on a stable patient*.

What is the major learning point?

*The initial management of patients with lung evisceration is critical, including standard trauma evaluation and resuscitation and appropriate airway management*.

How might this improve emergency medicine practice?

*Heightened awareness of this presentation is imperative to ensure that clinicians provide appropriate treatment*.

Initial management included fluid resuscitation with one liter of normal saline (NS), analgesia with ketoprofen and tramadol intravenously, and oxygen facemask at six liters per minute. His blood pressure improved, and he did not require blood products or intubation in the ED. The eviscerated lung portion was left in place and covered with saline-saturated sterile towels. A portable radiograph machine was not immediately available, and CT was not performed because of concerns for clinical instability occurring while in CT. Instead the patient was assessed by the trauma team, which recommended immediate surgical intervention.

**Image 3:**
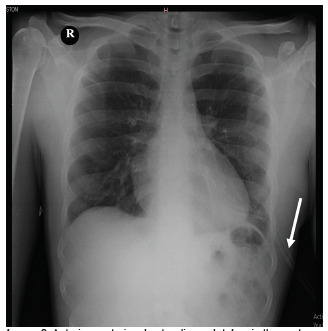
Anterior-posterior chest radiograph taken in the post-operative period for confirmation of the lung expansion. The chest tube was still in place (arrow).

The patient was taken to the operating room (OR), and under general anesthesia the chest was explored using a left anterolateral thoracotomy at the fifth ICS. Injury to the heart, great vessels, and diaphragm was ruled out; surgical exploration revealed only a small amount of hemothorax. The left lung had no signs of laceration, necrosis, or thrombosis. The eviscerated portion of the lung was determined to be the lingula. After confirming the integrity of the vasculature, the lingula of the lung was retrieved back into the left hemithorax, the left hemithorax was irrigated with two liters of NS, and the patient was ventilated by the anesthesia team to assess the anatomic integrity of the parenchyma. Once the lung showed no signs of air leakage during the insufflation test, the NS irrigation and secretions were suctioned, and closure of the left thoracotomy incision was performed. A thoracostomy tube was placed in the left hemithorax and attached to underwater seal drainage. After 48 hours, a chest radiograph was obtained for evaluation of re-expansion of the lung (Image 3).

The patient was kept under observation for 12 hours in the postoperative care unit and spent the rest of his hospital stay in the general surgery ward, not requiring intermediate or intensive care unit admission. He received tramadol for analgesia, prophylactic antibiotics with ampicillin-sulbactam three grams intravenously every six hours for seven days as an in-patient, and then continued with amoxicillin-clavulanic acid 875/125 milligrams orally every 12 hours for three days upon hospital discharge. He had an uneventful postoperative course, with a fully expanded lung on postoperative radiograph and steady functional and cosmetic improvement at 15- and 30-day follow-up outpatient visits.

## DISCUSSION

Evisceration of the lung is a rare and life-threatening injury. Patients presenting with injuries this uncommon may encounter inappropriate treatment because clinicians have little to no experience with this injury; so increasing awareness of the presentation and treatment of lung evisceration is imperative. The mechanism of injury can be penetrating or blunt. Blunt trauma mechanisms were reported in three cases with high-energy (motorcycle accident) mechanisms and resulting penetrating injuries to the thorax, possibly from open rib fractures.^2^,^3^,^5^ Our patient, like four others in published case reports, had stab wounds. All these patients were in stable condition (Table), with presenting complaints of chest pain and shortness of breath.

Although having a large chest wall defect may lead to open pneumothorax and respiratory distress, the presence of eviscerated lung can mechanically close the defect, preventing the passage of air and allowing the maintenance of normal ventilatory mechanics. A hemopneumothorax may be present, but the lung can still expand inside the hemithorax with each breath. Our patient’s presentation is concordant with the four previous reported cases that share the same mechanism of injury.^1^,^4^,^6^

Due to the limited number of cases, there are no universal guidelines regarding initial management of these patients. Therefore, the initial management upon patient presentation follows the standard trauma protocols with attention to oxygenation, perfusion, and hemorrhage control. In the majority of the cases where the patients were reported in stable condition, including this case, basic airway management was enough to maintain proper levels of oxygenation. However, if respiratory distress is suspected from a hemopneumothorax, tube thoracostomy should be performed. Furthermore, endotracheal intubation should be considered if relief of a tension pneumothorax does not improve the patient’s respiratory status, or if multi-system trauma or depressed mental status are present. Some patients, despite having large eviscerations, were in stable condition and definitive airway management occurred in the operating room.^1^,^3^,^4^,^6^ However, when respiratory distress was present, endotracheal intubation was performed in the ED.^2^

An important point of discussion is the appropriate time for endotracheal intubation, specifically whether it should be performed in the ED or OR. For example, a stable patient not requiring intubation for other reasons and going directly from the ED to OR may be managed safely by delaying intubation until OR arrival. However, if the patient needs to go to CT or elsewhere prior to the OR, or if there are delays in OR availability, then intubation in the ED may be appropriate since these patients can deteriorate at any time. In addition, positive pressure ventilation of an injured lung may affect the seal created by the eviscerated lung parenchyma and potentially create a tension pneumothorax or hemothorax; so endotracheal intubation may be considered in conjunction with tube thoracostomy of the affected side. These factors have to be considered especially if the patient requires transport to another facility, such as a trauma center, or from a battlefield.

In addition to recommendations on airway management, we also support an existing practice style to maintain the eviscerated lung in its position, without disturbance, if respiratory function is adequate and no air leak through the thoracic wall is detected. The eviscerated lung portion can block the passage of air through the thoracic wall defect, protecting the patient from massive pneumothorax and respiratory distress.^6^ Furthermore, the eviscerated lung may tamponade hemorrhage from a lacerated internal thoracic artery.^1^,^3^ Therefore, in a stable patient, do not attempt to replace the eviscerated lung back into the thorax but instead just cover it with saline-dampened sterile gauze or towels to keep the tissue viable. Leaving the eviscerated lung in place can be lifesaving, until it can be safely retracted into the chest in the OR under sterile and controlled conditions.

Some of the studies to consider before transport to the OR include a radiograph to evaluate for hemopneumothorax and FAST exam to evaluate for concurrent cardiac or intra-abdominal injuries. An E-FAST protocol (“E” stands for extended), which incorporates two views of the anterior thorax and allows the screening for pneumothorax with high sensitivity, may be preferred to FAST. In this case, the ultrasound operator was not trained in thoracic views, and so they were not performed to evaluate for pneumothorax. When the stability of the patient allows, a CT may be preferred to provide information about the size of the chest wall defect and associated injuries in the thorax and abdomen.^1^–^6^ However, these patients are at high risk of serious internal injuries and decompensation, and delay of definitive operative treatment can be detrimental.

Patients with penetrating thoracic injuries are at high risk of death due to injury of vital organs and great vessels. Furthermore, when lower chest (below the level of the nipples) stab wounds occur there is an increased risk of diaphragmatic and intra-abdominal injury. Additionally, hemorrhage may be significant if the internal thoracic or intercostal arteries are injured, which has been associated with lung evisceration and has a mortality rate of 40%.^15^ In this case the patient sustained a penetrating injury to the chest at the ninth ICS, which is not only concerning for intrathoracic injuries but also intra-abdominal injuries. During the operative exploration, lung, cardiac, vessels, and diaphragmatic injuries were ruled out. Only a small amount of hemothorax was found, which was attributed to intercostal muscle lacerations that were tamponaded by the lingula evisceration. After the integrity of organs and vessels was confirmed, the trauma team proceeded with the evisceration repair and sutured the superficial wound on the right hemithorax. The patient recovered uneventfully.

Most of the patients reported in the literature had good functional and aesthetical outcomes, as did our patient. The complications these patients may have include chronic chest pain and possible lung hernias.^10^ Herniation occurs as a result of injured intercostal muscles and sudden or sustained increases in intrathoracic pressure. Anterior herniation is more common than lateral herniation due to lateral reinforcement of the thoracic wall by the serratus muscle.^9^,^10^ Therefore, these patients must seek medical care in the event of new chest pain, shortness of breath, or chest wall deformities.

## CONCLUSION

The proper initial management of patients with lung evisceration is critical, including standard trauma evaluation and resuscitation, appropriate airway management, and not reducing the eviscerated lung. Prompt surgical exploration is important to retrieve and replace the lung back into the hemithorax, evaluate for associated injuries, and repair the chest wall defect.

## Figures and Tables

**Table:** Reported cases of lung evisceration in the literature.

Case report	Mechanism of trauma	Presentation	Airway management	Site of injury	Vessel injury	Surgical access	Hospital stay (days)	Outcome
Bowely^1^	Penetrating stab wound	Stable	Intubation in the OR	Left fourth, fifth and sixth intercostal joints	ITA	Anterior lateral thoracotomy	10	Lost to follow up
Lang-Lazdunsi^2^	Blunt/penetrating (MCA)	Respiratory distress	Intubation in the ED	Left clavicle, first, second and third ribs	Intercostal vessels	Transcostal vertical thoracotomy	26	One year good cosmetic result
de Leo^3^	Blunt/penetrating (MCA)	Stable	Intubation in the OR	Sternum, right clavicle and first to fourth ribs	ITA	Anterior thoracotomy	8	Two year good cosmetic result
García Toral^4^	Penetrating stab wound	Stable	Intubation in the OR	Left second to seventh costal cartilages	Not reported	Via stab	5	Six month good cosmetic result
David^5^	Blunt/penetrating (MCA)	Not reported	Not reported	Right clavicle, right second and third rib fracture	None	Via defect	10	Two month good cosmetic result
Suman Mewa Kinoo^6^	Penetrating stab wound	Stable	Intubation in the OR	Left second, third and fourth costal cartilages	Intercostal vessels	Via stab	6	One month good cosmetic result
Suman Mewa Kinoo^6^	Penetrating stab wound	Stable	Intubation in the OR	Left second and third costal cartilages	Intercostal vessels	Via stab	5	Lost to follow-up
Our report	Penetrating stab wound	Stable	Intubation in the OR	Left hemithorax, ninth ICS	None	Anterior lateral thoracotomy	7	One month, good functional – cosmetic result

*MCA*, motorcycle accident; *OR*, operating room; *ED*, emergency department; *ICS*, intercostal space; *ITA*, internal thoracic artery.

## References

[1] Bowley DM, Boffard KD (2001). Penetrating lung hernia with pulmonary evisceration: case report. J Trauma.

[2] Lang-Lazdunski L, Bonnet PM, Pons F (2002). Traumatic extrathoracic lung herniation. Ann Thorac Surg.

[3] de Leo S, Patriti A, Boselli C (2004). Traumatic evisceration of the lung without pneumothorax. Eur J Trauma.

[4] García TR, Torrano ME, Medel LB (2008). Successful delayed repair of lung evisceration secondary to stab wound. Rev Inst Nal Enf Resp Mex.

[5] David JS, Tassin C, Maury JM (2013). Post-traumatic pulmonary hernia. Thorax.

[6] Mewa Kinoo S, Ismail SB, Naidoo R (2017). Lung evisceration following penetrating chest trauma. Indian J Thorac Cardiovasc Surg.

[7] Wani AS, Kalamkar P, Alhassan S (2015). Spontaneous intercostal lung herniation complicated by rib fractures: a therapeutic dilemma. Oxf Med Case Reports.

[8] Hiscoe DB, Digman GJ (1955). Types and incidence of lung hernias. J Thorac Surg.

[9] Ishibashi H, Hirose M, Ohta S (2007). Lung hernia after video-assisted thoracoscopic lobectomy clearly visualized by three-dimensional computed tomography. Eur J Cardiothorac Surg.

[10] Arslanian A, Oliaro A, Donati G (2001). Posttraumatic pulmonary hernia. J Thorac Cardiovasc Surg.

[11] Emberger JS, Racine L, Maheshwari V (2011). Lung hernia associated with hemothorax following cardiopulmonary resuscitation. Respir Care.

[12] Brock MV, Heitmiller RF (2000). Spontaneous anterior thoracic lung hernias. J Thorac Cardiovasc Surg.

[13] François B, Desachy A, Cornu E (1998). Traumatic pulmonary hernia: surgical versus conservative management. J Trauma.

[14] Choe CH, Kahler JJ (2014). Herniation of the lung: a case report. J Emerg Med.

[15] Ritter DC, Chang FC (1995). Delayed hemothorax resulting from stab wounds to the internal mammary artery. J Trauma.

